# Spatial patterns of oxygen states affect lake food web structure through the response of cold-water fish

**DOI:** 10.1371/journal.pone.0354270

**Published:** 2026-08-18

**Authors:** Ryosuke Katayose, Ayato Kohzu, Natsuko Ito Kondo, Kayoko Fukumori, Munemitsu Akasaka, Taku Kadoya

**Affiliations:** 1 Biodiversity Division, National Institute for Environmental Studies, Tsukuba, Ibaraki, Japan; 2 United Graduate School of Agricultural Science, Tokyo University of Agriculture and Technology, Fuchu, Tokyo, Japan; 3 Regional Environment Conservation Division, National Institute for Environmental Studies, Tsukuba, Ibaraki, Japan; 4 Institute of Agriculture, Tokyo University of Agriculture and Technology, Fuchu, Tokyo, Japan; 5 Institute of Global Innovation Research, Tokyo University of Agriculture and Technology, Fuchu, Tokyo, Japan; University of Limpopo, SOUTH AFRICA

## Abstract

Surface water warming and bottom water oxygen depletion, accelerated by global warming, limit the distribution of cold-water fish in the pelagic areas of temperate lakes. While surface warming is well known to cause drastic changes to food web structure by limiting the distribution of top predators, the effect of hypoxia on cold-water fish distribution and food webs are lacking because hypoxic conditions are often spatially heterogeneous. We investigated how the spatial development of dissolved oxygen conditions is associated with the distribution of cold-water fish (kokanee and rainbow trout) and their prey resources. To address this, we compared the spatial distribution of cold-water fish and their prey resources between two pelagic sites during summer in a small temperate lake characterized by spatially heterogeneous dissolved oxygen conditions associated with its unique topography. We found that eDNA copies of kokanee were concentrated in areas with relatively favorable dissolved oxygen concentrations during the summer, when their vertical distribution was strongly constrained by low oxygen concentrations and high temperatures. These findings suggest that spatial heterogeneity in dissolved oxygen conditions can alter the pelagic distribution of cold-water fish during summer. Furthermore, changes in the distribution of cold-water fish potentially contributing to spatial variation in predation pressure, influencing the spatial distribution of zooplankton. These patterns are consistent with the possibility that spatial variation in fish distribution contributed to differences in predation pressure.

## Introduction

Global climate change stresses pelagic lake ecosystems by increasing the frequency and intensity of surface-water warming and simultaneously increasing bottom water oxygen depletion events [1–3]. The oxythermal habitat suitable for cold-water fishes such as salmonids is therefore diminished vertically by high surface temperatures and low bottom water oxygen concentrations [4]. Because some species of salmonids are top predators in limnetic ecosystems, changes in their distribution can influence ecological interaction within lake pelagic food webs [5–7]. Previous studies have mainly focused on the effects of surface water warming, particularly because a decrease in the access of cold-water fishes to littoral habitat forces them to stay in the pelagic zone [6]. However, dissolved oxygen conditions often vary spatially within lakes, potentially generating heterogeneous distributions of cold-water fishes even within the pelagic zone. Such spatial heterogeneity in predator distribution alter local predator–prey interactions and energy pathways within pelagic ecosystems, suggesting that dissolved oxygen heterogeneity may play an important role in shaping pelagic food-web structure.

While warming of surface waters tends to happen spatially homogeneous manner in the pelagic zone, the distribution of hypoxic conditions in bottom waters can be highly heterogeneous [8]. This heterogeneity of oxygen concentrations causes the extent of the vertical limitation of the oxythermal habitat suitable for cold-water fishes to vary across the pelagic zone. In fact, cold-water fishes that live in the pelagic zone move horizontally to escape hypoxic bottom waters [9] and to concentrate in waters with more favorable oxygen concentrations [8,10]. In contrast, pelagic prey such as zooplankton generally has more limited horizontal mobility. The spatial aggregation of predators mediated by oxygen concentrations causes spatial differences in predation pressure that impact the distribution of zooplankton in the pelagic zone [8]. In other words, a site in the pelagic zone with high (low) oxygen concentrations will be associated with a high (low) density of cold-water fish, which may exert more (less) predation pressure on zooplankton. The result is a relatively low (high) density of zooplankton. These localized differences in oxygen concentrations and the distribution of cold-water fish within the pelagic zone can thus lead to emergent large-scale patterns of spatial heterogeneity in the strength of top-down effects at the lake-wide level. However, how cold-water fish respond to spatially heterogeneous oxygen concentrations both horizontally and vertically in the pelagic zone and how their response cascades down through the pelagic food web have rarely been assessed.

**Fig 1:**
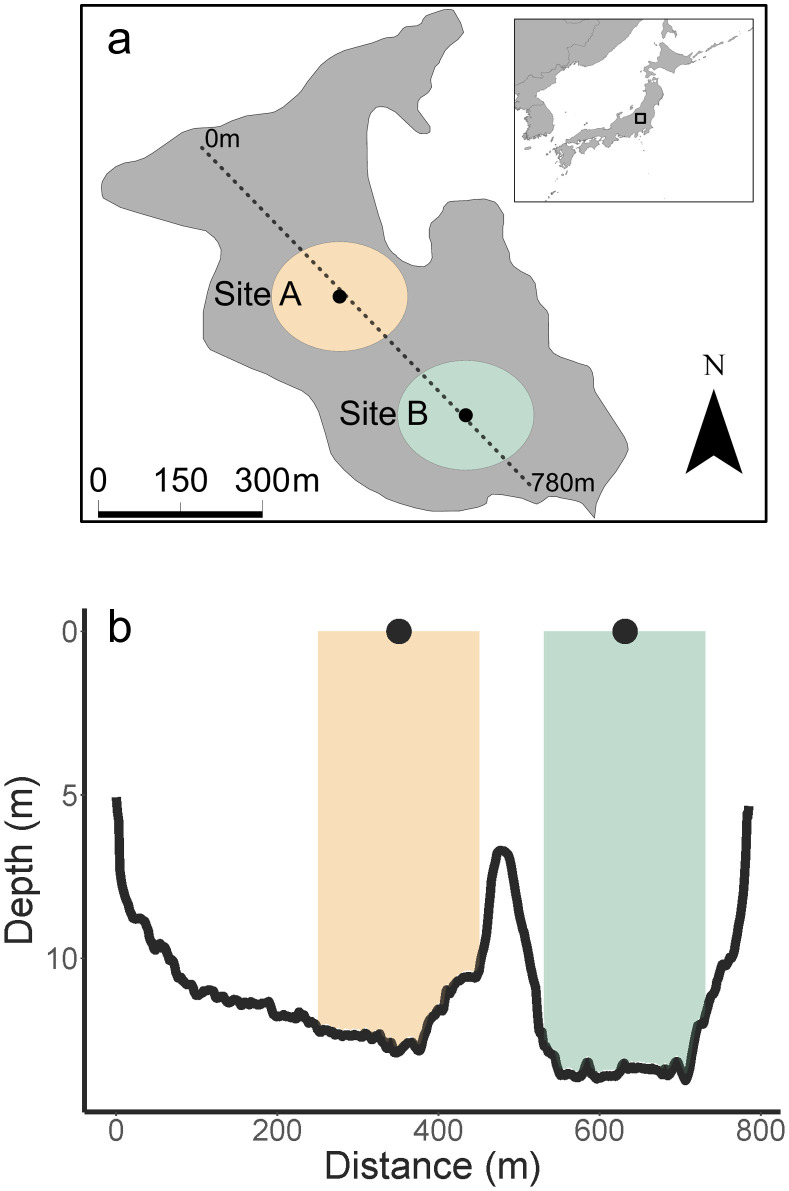
Overview of topographic conditions at Lake Yunoko. a, Location of Lake Yunoko and survey sites. The census survey was conducted using a hydroacoustic system in the area defined by the 100-m radius of the two colored areas (Site A and B).The map was generated using ArcGIS Pro 2.9.5 (Environmental Systems Research Institute, Redlands, CA, USA; https://support.esri.com/en-us/patches-updates/2022/arcgis-pro-2-9-patch-5-2-9-5-announcement-8071) with lake surface data from the National Land Numerical Information (MLIT, Japan) and administrative boundary data from the Comprehensive Global Administrative Zones (CGAZ), both distributed under the Creative Commons Attribution 4.0 International (CC BY 4.0) license. b, The intra-lake topography along the dotted line in panel a. The ridge topography divides the lake into two basins.

Here, we addressed this knowledge gap by examining the response of the distribution of cold-water fish species to the seasonal dynamics of dissolved oxygen concentrations and water temperature (Fig 1A). In particular, we selected a temperate lake with distinctive topographic features that were expected to generate spatial heterogeneity in dissolved oxygen conditions as a case study system (Fig 1B), and spatial comparisons within the lake were interpreted as within-lake contrasts. To comprehensively characterize the spatial distribution of a specific cold-water fish species in the pelagic zone, we combined environmental DNA (eDNA) techniques, which captured the relative spatial distribution of the cold-water fish species, with hydroacoustic surveys, which enabled us to estimate the distributions of the populations of fish communities at two study sites as a within-lake spatial contrast (Site A and Site B, Fig 1A). In addition, to discuss the potential propagation of top-down cascading effects associated with spatial heterogeneity of the distribution of cold-water fish, we explored the relationships between the density of cold-water fish, dissolved oxygen concentrations, zooplankton densities, chlorophyll-a concentrations, and water transparency. We tested the following hypotheses: (1) whether the distribution patterns of cold-water fish vary in response to spatially heterogeneous dissolved oxygen conditions, and (2) whether corresponding within-lake distribution patterns of zooplankton and phytoplankton emerge in association with spatial heterogeneity in cold-water fish distribution.

## Materials and methods

### Study site

The surveys were conducted monthly from June to November 2022 at Lake Yunoko in Japan (36.80°N, 139.43°E). Lake Yunoko has a maximum depth of 13.6 m and a surface area of 0.32 km^2^ (Fig 1A). The lake is divided into north and south basins by a topographic ridge at a depth of around 6.7 m. The maximum depths are 12.2 m in the north and 13.6 m in the south (Fig 1B). Because we suspected that water quality might differ between the north and south basins, we sampled at Site A (north side) and Site B (south side) near the deepest parts of the lake (Fig 1B). All surveys were conducted during daytime on the same day. The vertical water quality surveys, eDNA sampling, phytoplankton biomass survey, and zooplankton sampling were carried out between 11:00 and 14:00, followed by hydroacoustic surveys conducted before sunset. Permissions to conduct the field surveys were obtained from four authorities: the Kanto Regional Environmental Office (Ministry of the Environment), the Fisheries Technology Institute (Japan Fisheries Research and Education Agency), the Nikko Civil Engineering Office (Tochigi Prefectural Government), and the Nikko City Government.

### Target fishes

In this survey, we targeted kokanee (*Oncorhynchus nerka*) and rainbow trout (*Oncorhynchus mykiss*). Kokanee and rainbow trout are Salmonidae, and suitable water temperatures for their respective habitats are below 13 °C [11,12] and 17 °C [13]. The suitable dissolved oxygen concentration for their habitats is 2 mg/L or higher [14–17]. Some cold-water fish species are released annually in Lake Yunoko. The particularly large numbers of kokanee and rainbow trout that are released into this lake each year amount to approximately 6000 kg. The fish fauna in the pelagic zone is therefore almost entirely dominated by salmonids [18].

### Detection of spatial distribution of the fish communities

Census surveys were conducted monthly using a hydroacoustic system and a vessel to quantify fish density by depth at each survey site. Because the hydroacoustic system could provide information on the positions of individual fish, it allowed for an accurate understanding of the vertical distribution of fish assemblages. Fish detection was conducted by a powered vessel equipped with two hydroacoustic systems (Deeper Sonar, Vilnius, Lithuania) to obtain a sufficient number of repetition that made three round trips at an average speed of 3.5 km/h in a north-south direction around the water quality study sites. Sampling was repeated a total of 12 times. To account for the variability of fish detection, routes were set at intervals of approximately 20 m, and the central route was navigated directly above the water quality study points. Detection was conducted within a radius of 100 m of each of the two study sites to collect data on the vertical distribution of fish at each survey site. The depth at which individuals were detected was recorded for each route, and a total of 4902 individuals were recorded during the monthly surveys from June to November. Because the search area widened conically with depth (20^o^), the search volume was calculated for each 1 meter of water depth, and the fish density was calculated as a function of depth by dividing the number of detected individuals by the search volume.

### Detecting the distribution of salmonid fish using eDNA

The eDNA approaches have been widely applied to assess fish distributions in aquatic ecosystems [19], and their spatial and temporal resolution has been clarified. Horizontal eDNA is known to dispersal within approximately 100 m, although the exact spatial resolution depends on lake size and other environmental conditions in lentic freshwater ecosystems [19]. Vertical eDNA distributions have also been shown to partially reflect the development of thermal stratification and hypoxic conditions [18,20]. In terms of temporal resolution, validation studies in marine systems demonstrated that eDNA signals can persist for approximately two hours [21]. Taken together, these findings suggest that monthly snapshot surveys using eDNA can adequately capture the horizontal distributions of cold-water fishes between Site A and Site B.

In this study, the relative distribution of salmonid fish species between sites was quantified using eDNA. Six-liter water samples were collected at depths of 3, 5, 7, 9, and 11 m using a Van Dorn water sampler (RIGO Co.Ltd, Tokyo, Japan), and a sample of 1 L was stored.

To prevent DNA degradation, water samples were immediately fixed with 1 mL of Osvan S solution (Nihon Pharmaceutical, Tokyo, Japan) to achieve a final benzalkonium chloride concentration of 0.01% [22] and kept on ice until filtration. Samples were filtered through Whatman 47-mm-diameter GF/F glass microfiber filters (Cytiva, Tokyo, Japan) within 24 hours of collection. One-liter water samples were filtered using two GF/F filters, except for the sample collected at a depth of 9 m at Site A in September, which was filtered using three filters. For the filtration blank sample, 1 L of ultrapure water (MilliQ, Millipore) was filtered on each collection day. The filters were wrapped in aluminum foil and stored at –28 °C until DNA extraction. DNA was extracted using the DNeasy Blood and Tissue Kit (QIAGEN, Hilde, Germany). Each filter was incubated with Buffer AL and Proteinase K at 56 °C; lysates from the same 1-L water sample were pooled and processed using a single spin column. DNA was eluted in a final volume of 100 μl per 1-L water sample. One negative control was prepared for each day of DNA extraction to monitor potential cross contamination.

To quantify the relative distribution of two salmonid species, qPCR analysis of eDNA was performed using species-specific primers and probes for kokanee salmon [18] and rainbow trout [23]. Each qPCR reaction was performed in a total volume of 20 μL, containing 1×TaqMan Environmental Master Mix 2.0 (Thermo Fisher Scientific), 900 nM of each primer, 125 nM of the TaqMan probe, and 2 μL of template eDNA. Thermal cycling was conducted on a LightCycler 480 (Roche, Mennheim, Germany) under the following conditions: initial denaturation at 95 °C for 10 min followed by 50 cycles of 95 °C for 15 s and 60 °C for 60 s. Each sample was analyzed in triplicate. Copy numbers were estimated from a standard curve generated using a six-point serial dilution ranging from 10 to 1 × 10^−6^. No-template and negative controls were consistently included in all qPCR reactions to detect potential contamination. The average copy number across three replicates was calculated for each sample and converted to copies per liter of water. All negative controls, including those for filtration, DNA extraction, and qPCR, resulted in no amplification in any qPCR assay.

### Detection of vertical water quality, water temperature, and transparency

Water quality and water temperature were observed at 1-m intervals using ProDSS (YSI, Yellow Springs, Ohio, USA) at the study sites. The observed parameters included water temperature, dissolved oxygen, turbidity, conductivity, pH, oxidation–reduction potential, and chlorophyll-a concentrations as a metric of phytoplankton biomass. Secchi depths were used as a metric of water transparency.

### Zooplankton sampling

Vertical profiles of zooplankton abundance were collected at two study sites. Water samples were collected at the surface (1 m), mid-depth (5 and 7 m), and bottom (11 m) using a Van Dorn water sampler (6 L). The samples were strained through a net (79-μm mesh), fixed in sugar formalin, and stocked. Zooplankton were identified using a microscope, and the number of individuals of each species was measured. The population density (individual/L) was calculated for 12 species based on the ratio of the volume of the entire sample to the volume of the species (S4 Fig). We used Konno and Sakano (2010) [24] as a reference to determine the species that kokanee prey on (*Daphnia galeata, D. ambigua*, and *Cyclopoida sp.*) and other species (*Bosmina sp., copepodid of Cyclopoida, nauplius of Copepoda, Asplanchna sp., Keratella cochlearis, Keratella quandrata, Polyarthra sp., Synchaeta sp.*, and *Filinia longiseta*).

### Statistical analysis

We compared fish densities within and outside suitable oxythermal habitats to assess whether high temperatures and low dissolved oxygen concentrations limited the distribution of fish. The thresholds observed via field surveys and experiments for high temperature and low dissolved oxygen concentration of kokanee were less than 13 °C [11,12] and more than 2 mg/L of dissolved oxygen [14–17], respectively. The area with water temperatures below 13 °C and dissolved oxygen concentrations above 2 mg/L was defined to be suitable oxythermal habitat for kokanee at Lake Yunoko. Because suitable oxythermal habitats were observed from July to October at both Site A and Site B, analyses of fish distribution were conducted during that period. The fish densities observed within and outside the suitable oxythermal habitat were compiled as a function of depth, and Bayesian estimation of fish density and density difference between within and outside the suitable oxythermal habitat was conducted for each month and for each of the two sites. Specifically, we assumed that the fish density plus 0.01 followed a log-normal distribution, and we sampled the posterior distribution using the Markov Chain Monte Carlo (MCMC) method with the Just Another Gibbs Sampler (JAGS). Bayesian estimation of density difference of zooplankton that prey for kokanee between sites was also conducted for each month. We assumed that the density difference of these zooplankton followed a normal distribution, and we sampled the posterior distribution using the MCMC method with JAGS. The 95% credible interval calculated from the posterior distribution was used to evaluate whether there was a difference between groups. A difference between groups was considered to exist if the credible intervals did not include zero. Convergence of the model was confirmed when the *R*^2^ values of all variables were less than 1.1.

To test for differences in detected eDNA concentrations between sites, we compared eDNA copies for each month from June to November using a generalized additive model, assuming that eDNA copies followed a negative binomial distribution as the response variable, and we constructed a model with month as the explanatory variable for each of two sites for kokanee and rainbow trout. Simulation-based residual diagnostics using the DHARMa package [25] indicated deviations from the expected residual distribution in one GAM, including evidence of underdispersion and deviations in the QQ plot. However, the estimated smooth term showed low complexity (EDF ≈ 1), no strong systematic structure was observed in residual patterns, and the main trends remained qualitatively consistent across alternative model formulations. These results suggest that the overall conclusions were robust despite some limitations in model fitness. The mgcv package in R ver 4.4.1 [26] was used for the analyses. We used R ver 4.4.1 statistical software for all statistical analyses.

## Results

**Fig 2:**
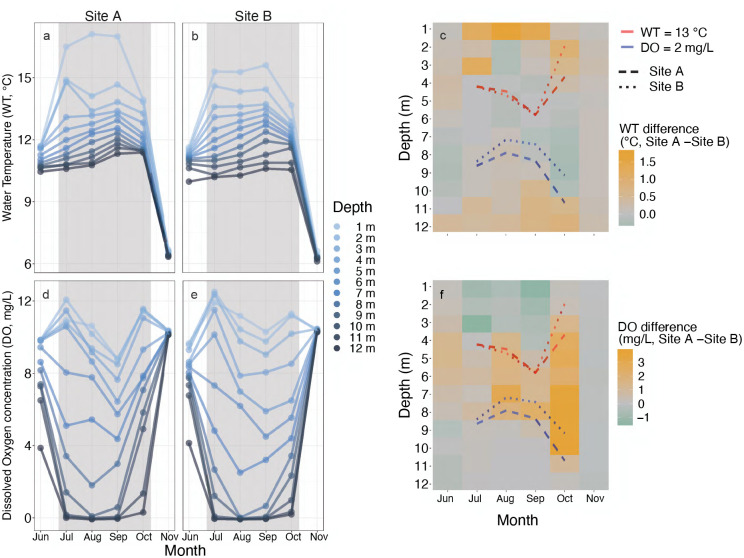
Vertical profile of water temperature and dissolved oxygen concentration by site and month. a, b, d, and e, Gray shading indicates months when observed water temperature exceeded 13 °C and dissolved oxygen (DO) concentrations fell below 2 mg/L. c and f, The heat map represents the difference of water temperatures and dissolved oxygen concentrations at the same depth between sites A and B. Blue dotted and dashed lines indicate depths with DO of 2 mg/L, and red dotted and dashed lines indicate depths at which the water temperature was 13 °C.

Temperatures exceeding the suitable temperature threshold of rainbow trout (*Oncorhynchus mykiss*) (17 °C [13]) were observed only at a depth of 1 m (Fig 2A and Table 1). The vertical range of the depths of suitable oxythermal habitat for Kokanee was consequently reduced to as little as 1.68 m (depths of 5.79–7.47 m) (S1 Table). Comparison of water quality parameters between sites at the same depth from July to October, when suitable habitat for cold-water fish was restricted, revealed no clear differences in temperature, oxidation–reduction potential, turbidity, electric conductivity, or pH between sites (Fig 2C and S1 Fig). However, dissolved oxygen concentrations were higher at Site A and were within the oxythermal habitat suitable for kokanee (Fig 2F).

**Table 1:** Summary of vertical profiles of water quality, water temperature, and chlorophyll-a concentration measured once a month from June to November.

Variable	Description	Site	Mean	Median	SD	Max	Min
WT	Water temperature (°C)	A	11.35	11.61	2.61	17.11	6.33
		B	11.12	11.43	2.49	15.61	6.11
DO	Dissolved oxygen concentration (mg/L)	A	7.00	8.56	3.97	12.06	0.00
		B	6.40	7.84	4.24	12.50	0.00
Turbidity	Turbidity (FNU)	A	1.42	1.06	1.24	6.13	0.06
		B	1.19	1.07	0.88	4.93	0.18
ORP	Oxidation–reduction potential (mV)	A	98.21	99.05	53.62	179.00	–62.80
		B	107.48	117.30	82.99	220.30	–134.10
pH	pH	A	6.81	6.86	0.46	7.94	6.02
		B	6.80	6.68	0.45	7.96	6.15
EC	Electrical conductivity (µS/cm)	A	171.11	162.10	26.42	249.80	121.50
		B	165.57	166.60	15.02	195.50	133.30
Chloro	Chlorophyll-a concentration (µg/L)	A	6.32	4.66	4.64	19.76	0.59
		B	7.11	6.24	4.72	21.15	0.98

**Fig 3:**
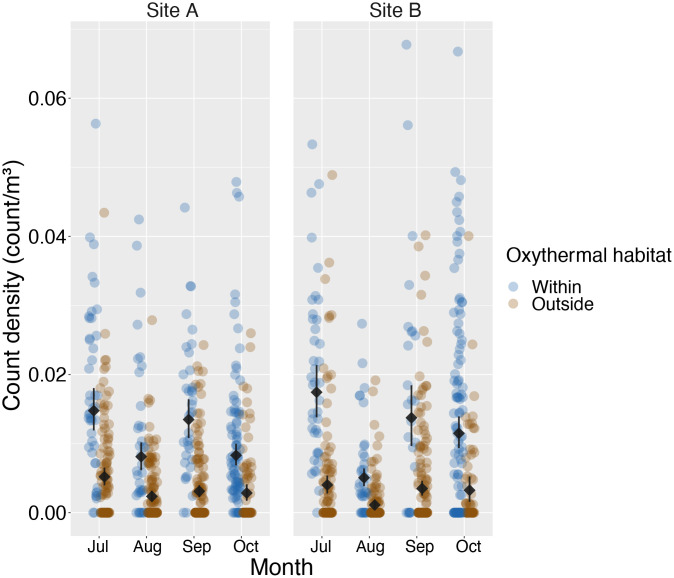
Comparison of fish density between suitable oxythermal habitats (water temperature < 13 °C and DO ≥ 2 mg/L) and other areas for kokanee. Diamonds and vertical bars represent the median and 95% credible intervals of estimated fish density (see S1 Table).

We examined whether high temperatures and low dissolved oxygen concentrations limited the vertical distribution of the fish. Fish densities were compared between within and outside the areas of suitable oxythermal habitat for Kokanee using a hydroacoustic system on a vessel from July to October, when high temperatures at the surface and low oxygen concentrations at the bottom were observed (Fig 2). We found that the median fish density by depth was consistently higher within versus outside of the suitable oxythermal habitat at both sites from July to October (S2 Table and Fig 3).

**Fig 4:**
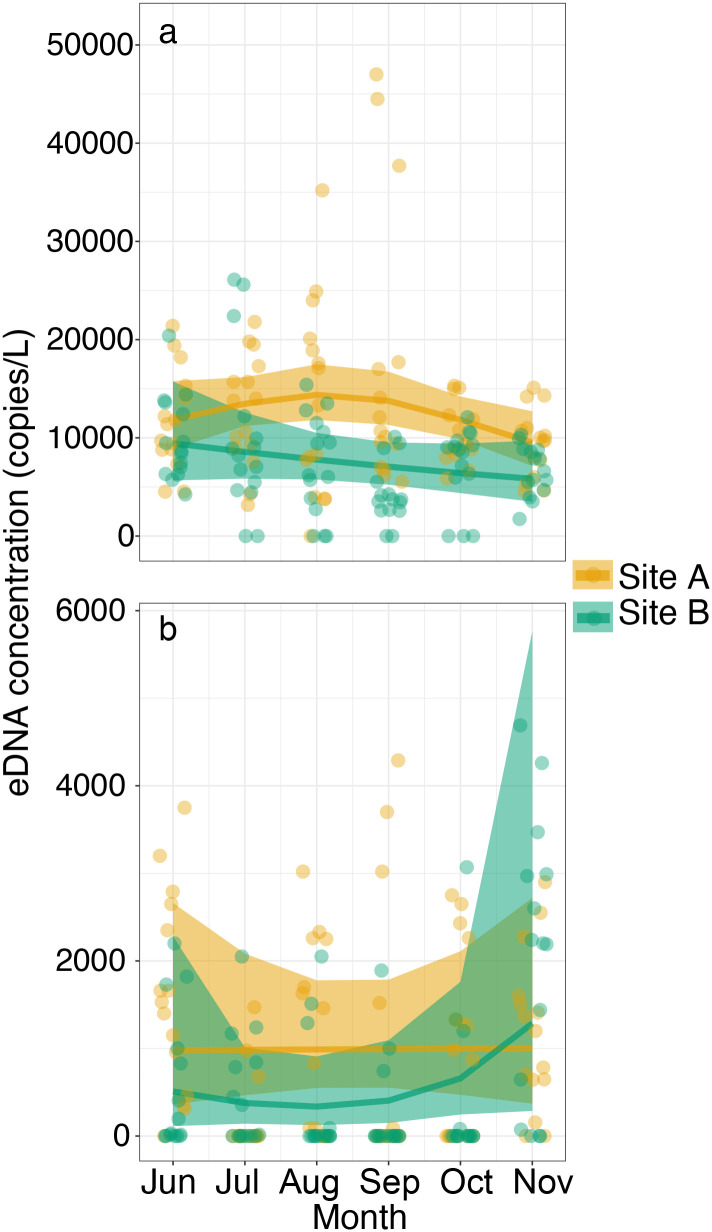
Measured eDNA concentrations compared among sites. Results are shown separately for each species using species-specific primers (a, kokanee; b, rainbow trout). The *x*-axis shows months, and the *y*-axis shows eDNA concentration. The line and shaded area represent median and 95% confidence intervals of the estimates. Depth-specific measurements were pooled by site and month, and site-specific generalized additive model estimates are indicated by orange (Site A) and green (Site B).

The quantitative PCR technique allowed us to assess the relative distribution of individual species based on spatial variations of eDNA [27]. The results showed that the eDNA concentration of kokanee was higher at Site A than at Site B from August to October, when dissolved oxygen concentrations were higher at Site A (Fig 4A). However, the eDNA concentrations of rainbow trout were not clearly different between sites (Fig 4B). Similarly, fish density within the thermal and dissolved oxygen thresholds did not differ clearly between sites from July to September. (S2 Fig, S3 Table).

**Fig 5:**
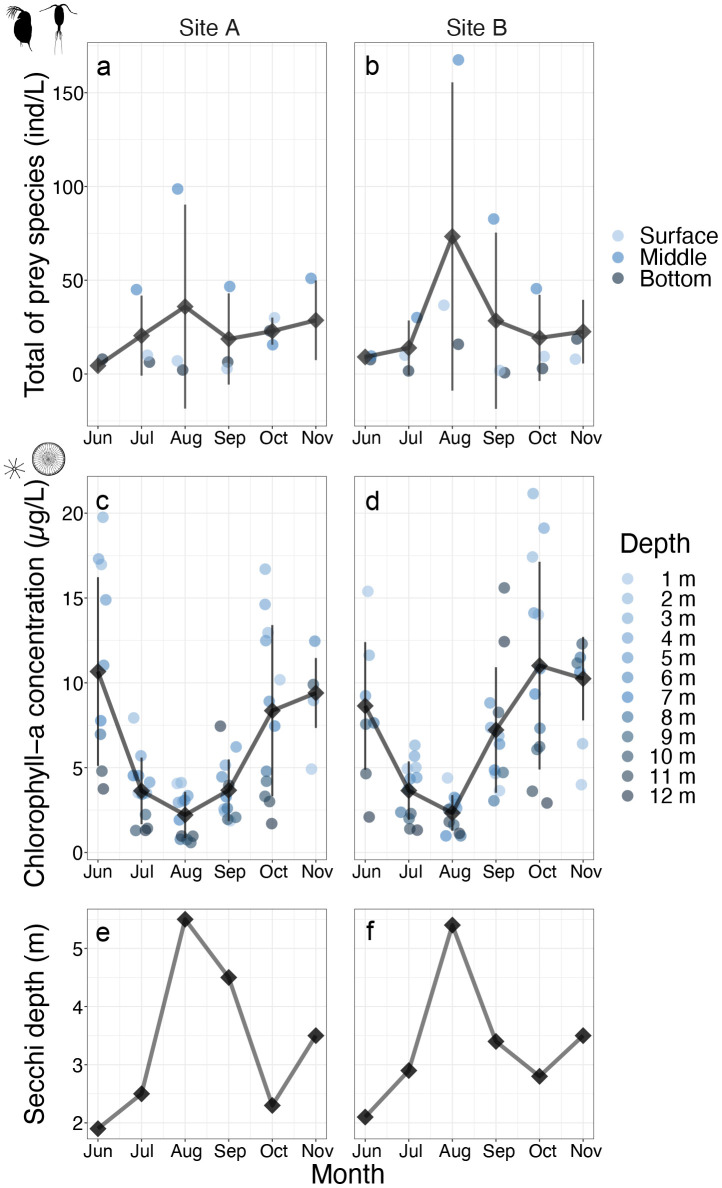
Monthly changes in zooplankton density, chlorophyll-a concentration, and water transparency at each site. a, b, Conditions from June to November for the amount of zooplankton to kokanee predation, c, d chlorophyll-a concentration, and e, f Secchi depth. In panels a–d, different colors indicate different sampling depths, and vertical bars represent variances.

We assessed zooplankton population densities, chlorophyll-a concentrations, and Secchi depths at Site A and Site B. The results showed that zooplankton population density peaked in August, when chlorophyll-a concentration and Secchi depths were lowest (Fig 5). Conversely, there was no clear difference in chlorophyll-a concentrations and Secchi depths between the sites throughout the survey period (Fig 5C, 5D, 5E and 5F). It is worth noting, however, that the density of zooplankton taxa that were categorized as potential prey of kokanee was up to 1.6 times higher in the middle layer from August to October at Site B, where the kokanee density was lower than at Site A, although the difference in density of the zooplankton taxa was not statistically significant (S3 Fig, S4 Table).

## Discussion

Because of the high surface water temperature during summer caused by global warming, the vertical distribution of cold-water fishes has shifted to deeper water, and that shift has altered the dependency on food resources from littoral to pelagic zones [6,7]. In the present study, we found that the horizontal heterogeneity of dissolved oxygen concentrations caused the distributions of cold-water fish to be patchy. This phenomenon will likely become more severe under future warming and may result in a decrease in spatial connectivity of cold-water fish and their zooplankton resources. In the case study of a temperate lake, the spatial heterogeneity in dissolved oxygen conditions would contribute to the spatial distribution of ecological interactions within the pelagic zone.

The spatial heterogeneity of dissolved oxygen concentrations in the lake and their relationship with topographic factors localized the horizontal distribution of kokanee by limiting their movement between sites. The fact that the dissolved oxygen concentrations at Site A were relatively high and within the oxythermal habitat suitable for kokanee allowed the kokanee to stay longer and reach higher population densities there than at Site B. Furthermore, the shape of the lake bottom and the high surface temperature of Lake Yunoko restricted the movement of kokanee between the two sites during the summer. Because the maximum depth of water temperatures above 13 °C was 5.79 m, the depth interval from that depth to the ridge topography (6.7 m in depth) separating Sites A and B was only about 1 m. Although careful interpretation is required when relating eDNA concentrations to spatial distribution patterns because eDNA signals can be influenced by physical processes such as water movement and diffusion, these findings suggest that kokanee had to pass through the high-temperature zone if they were to move between sites beyond the lake ridge topography during the summer. They further suggest that the horizontal heterogeneity of dissolved oxygen concentrations in the pelagic zone was as crucial as the development of low bottom-water oxygen concentrations in restricting the distribution of cold-water fish.

The phytoplankton biomass showed little difference between sites, whereas midwater zooplankton densities differed spatially during August to October. Midwater zooplankton, which are the major prey for kokanee, were more abundant at Site B from August to October, where eDNA concentrations of kokanee were lower than at Site A. These spatial patterns suggest a possible association between kokanee distribution and zooplankton density. Although, this pattern should be regarded as preliminary because differences in zooplankton density were not statistically significant, one possible explanation is that spatial variation in kokanee distribution influenced localized predation pressure on zooplankton. Indeed, kokanee feed primarily on mid-water zooplankton, whereas rainbow trout feed on benthos and insects from the bottom and surface [28]. Similar localized reductions of zooplankton in Lake Erie have been attributed to horizontal migration of some fishes to avoid hypoxic water [8]. In addition, hypoxic water can serve as a refuge for zooplankton because they can avoid predation by fish [29,30]. Although predation processes were not directly examined in this study, our observed spatial patterns are consistent with the hypothesis that spatial heterogeneity in dissolved oxygen conditions may influence localized predator and prey interactions within pelagic zone. Future studies using higher-resolution spatial observations, long-term observations and a comparison across multiple lakes would help clarify how environmental gradients shape organism distributions and ecological interactions within pelagic zone.

We detected no difference of the eDNA concentrations of rainbow trout between two sites. The similarity of the eDNA concentrations is reasonable because the highest temperature in Lake Yunoko did not exceed the physiological tolerance of rainbow trout; the fact that the highest temperature of Lake Yunoko was 17.1 °C (Table 1) at a depth of 1 m suggests that the thermal limitation of the habitat was weaker for rainbow trout than for kokanee. This result is consistent with the previous detection of the eDNA of kokanee in deep, cold waters, whereas the eDNA of rainbow trout was uniform throughout the water column of Lake Yunoko [18]. The greater tolerance of rainbow trout to high temperatures allowed them to use the near-surface layer, where dissolved oxygen concentrations were high. The restriction of the distribution of rainbow trout by the ridge topography on the lake bottom may not have severely limited their horizontal distribution. We should note, however, that the detected eDNA values of rainbow trout were approximately one-tenth those for kokanee, and we did not detect the eDNA of rainbow trout in 78 of 180 samples. Although the pattern of rainbow trout that we found was reasonable given their biology (vide supra), it is possible that the quantity of eDNA of rainbow trout was insufficient to detect spatial differences.

Our results suggest that habitat restriction caused by surface high temperatures and bottom hypoxic conditions during summer is associated with seasonal stratification dynamics and the spatial distribution of cold-water fish. Indeed, from July to October, when vertical restriction of fish distribution was observed, the development of thermal stratification and expansion of bottom hypoxia occurred alongside increasing surface water temperatures (Fig. 2). These seasonal dynamics in mixing conditions would have contributed to the observed spatial distribution of cold-water fish within the pelagic zone. Considering that previous studies have predicted that climate warming will strengthen and prolong thermal stratification [31] while also promoting the expansion of hypoxic waters [1], habitat restriction of cold-water fish may become increasingly severe and prolonged under future warming scenarios. Therefore, future studies on sustainable resource management and lake ecosystem conservation should focus on developing a better understanding of the long-term relationships among stratification dynamics, habitat availability, and population dynamics, in addition to observations of seasonal distribution patterns.

Our study, which was based on monthly snapshot observations during one year in a single lake, was limited by our ability to detect spatial differences in dissolved oxygen concentrations, and these differences shape pelagic distributions and interactions. To better examine the direct relationship between environmental changes and ecological responses, it is crucial to conduct high-frequency monitoring of water quality and temperature as a function of depth, together with validations based on cold-water fish behavior and their feeding preferences. In addition, because eDNA signals can be affected by transport, diffusion, degradation and detection limits, the spatial patterns observed in this study should be interpreted with caution. Future studies integrating eDNA surveys with direct observations of fish distributions and movements would help validate the ecological interpretation of eDNA-derived distribution patterns. Furthermore, our findings were derived from a lake with a bottom that was distinctively divided into two basins. As a result, additional studies across multiple lakes are required to generalize our findings into other lake systems. Because we conducted an observational survey, our analyses were inherently correlational. To more robustly infer causal relationships between the spatial heterogeneity of water quality and food web dynamics, future research should consider experimental designs such as Before-After-Control-Impact frameworks [32] that incorporate replication and control sites and/or multiple lakes, as well as more sophisticated statistical modeling approaches that could be applied to higher resolution data. Finally, both fish and zooplankton are known to perform diel vertical migration to improve foraging efficiency and reduce predation risk [33]. In addition, the intensity of zooplankton diel vertical migration is known to increase in the situation of fish presence [34]. Therefore, examining the nighttime distributions of both fish and zooplankton may provide further insight into the potential relationship between the differences in fish density and predation pressure, which could not be directly evaluated in this study. Those improvements of the research approach would advance our understanding of the spatial and temporal stability of suitable habitats and strengthen the conclusions from the present study that the spatial variations of dissolved oxygen concentrations are as crucial as temperature in determining the impacts of warming on lake ecosystems.

## Supporting information

S1 Fig Monthly observations of water quality parameters and differences between sites at corresponding depths. a, b, d, e, g, h, j, and k Observed water quality parameters (ORP, turbidity, electrical conductivity, and pH) at each site. c, f, i, and l Differences between sites at the same depth as the heat map (Site A – Site B).
(TIF)

S2 Fig Comparison of fish count volume between sites within oxythermal suitable habitat (WT < 13 °C and DO ≥ 2 mg/L), based on median estimates. Diamonds and vertical bars represent the median and 95% credible intervals of estimated fish density (see S3 Table).
(TIF)

S3 Fig Comparison of zooplankton density between sites by layer (Surface, Middle, Bottom). (TIF)

S4 Fig Monthly densities (individuals/L) of 12 zooplankton taxa, shown separately for three depths. The densities of three taxa (*D. galeata, D. ambigua, Cyclopoida sp.*), which are predated on by kokanee, are summarized in Fig 5.
(TIF)

S1 Table Monthly depths of 13°C water temperature (WT) and 2-mg/L dissolved oxygen concentration (DO), along with their depth difference between sites (width). (CSV)

S2 Table Comparison of fish density between oxythermal suitable habitat (Within; WT < 13 °C and DO ≥ 2 mg/L) and outside areas (Outside), based on median estimates. Differences between within and outside not overlapping zero are shown in bold. From July to October, fish density was consistently higher within than outside the oxythermal suitable habitat.
(CSV)

S3 Table Comparison of fish density between sites within oxythermal suitable habitat (WT < 13 °C and DO ≥ 2 mg/L), based on median estimates. Differences between within and outside not overlapping zero are shown in bold.
(CSV)

S4 Table Monthly estimates of differences in zooplankton density between sites (Site A – Site B). Results are shown for the total density of all species (Total of all species), the total density of species subject to kokanee predation (Total of prey species), and the total density of species that were not preyed upon by kokanee (Total of non-prey species).
(CSV)
